# *KRAS* and *BRAF* Mutations as Prognostic and Predictive Biomarkers for Standard Chemotherapy Response in Metastatic Colorectal Cancer: A Single Institutional Study

**DOI:** 10.3390/cells9010219

**Published:** 2020-01-15

**Authors:** Nuria Garcia-Carbonero, Javier Martinez-Useros, Weiyao Li, Alberto Orta, Nuria Perez, Cristina Carames, Tatiana Hernandez, Irene Moreno, Gloria Serrano, Jesus Garcia-Foncillas

**Affiliations:** 1 Translational Oncology Division, OncoHealth Institute, Fundacion Jimenez Diaz University Hospital, Av. Reyes Católicos 2, 28040 Madrid, Spain; nuria.garciac@quironsalud.es (N.G.-C.); weiyao.li@quironsalud.es (W.L.); 2 Oncology Department, OncoHealth Institute, Fundacion Jimenez Diaz University Hospital, Av. Reyes Católicos 2, 28040 Madrid, Spain; alberto.orta@quironsalud.es (A.O.);; 3 Pathology Department, Fundacion Jimenez Diaz University Hospital, Av. Reyes Católicos 2, 28040 Madrid, Spain; nperez@fjd.es; 4 START Madrid-FJD, Hospital Universitario Fundación Jiménez Díaz, Av. de los Reyes Católicos, 2, 28040 Madrid, Spain; tatiana.hg@fjd.es; 5 START Madrid-Hospital HM Sanchinarro, Calle de Oña, 10, 28050 Madrid, Spain; imorenocandilejo@gmail.com; 6 Oncology Department, University Hospital Infanta Leonor, Avenida de la Gran Vía del Este, 80, 28031 Madrid; Spain; gloriamaria.serrano@salud.madrid.com

**Keywords:** *KRAS*, *BRAF*, metastatic colorectal cancer, prognosis, chemotherapy, anti-EGFR, biomarker

## Abstract

*KRAS* mutation is a confirmed predictive biomarker for anti-EGFR monoclonal antibody therapy response for metastatic colorectal cancer. However, its prognosis impact and the predictive potential for first-line standard chemotherapy remains unclear. On the other hand, V600E mutation is the most frequent and studied mutation in the *BRAF* gene, and it has been associated with a poor outcome of patients and a low response to anti-EGFR treatment. Thus, the aim of this study is to evaluate the role of *KRAS* and *BRAF* mutations as prognosis factors and predictive biomarkers for 1st line standard chemotherapy in metastatic colorectal cancer. *KRAS* mutations and *BRAF* V600E mutations exhibited a poor outcome (*p* = 0.021 and *p* < 0.0001, respectively). Cox multivariate analysis showed that the presence of liver metastasis (HR = 1.595; 95% CI: 1.086–2.343; *p* = 0.017), *KRAS* mutation (HR = 1.643; 95% CI: 1.110–2.431; *p* = 0.013) and *BRAF* V600E mutation (HR = 5.861; 95% CI: 2.531–13.570; *p* < 0.0001) were statistically significant co-variables for progression-free survival. Interestingly, patients with *KRAS* mutations were associated with a poor response to first line standard chemotherapy (*p* = 0.008). In contrast, the *BRAF* V600E mutation did not have any impact on the first line standard chemotherapy response (*p* = 0.540). Therefore, in the present study, we provide new insight on the role of *KRAS* and *BRAF*, not only as prognosis biomarkers, but also as first line standard chemotherapy response biomarkers in metastatic colorectal cancer.

## 1. Introduction

Colorectal cancer (CRC) is the third most common tumor diagnosed worldwide [1]. The death rate of CRC has dropped by 53% since 1970, primarily due to new advances in early diagnosis and treatment strategies. The 5-year overall survival has increased by 7.9% in patients aged 65–74 years [2]. Nevertheless, it still constitutes the third leading cause of cancer-related death in the United States [1]. Despite the improvement in CRC diagnosis and treatment, approximately 35% of CRC patients show stage IV or metastatic disease (mCRC) at diagnosis, and 20–50% of stage II and III patients will progress to metastatic disease [3]. The most common sites of distant metastases in mCRC are liver (50–60%), lymph nodes (35–40%), lung (10–30%) and peritoneum (5–20%) [4]. Thus, the 5-year survival rate for metastatic disease is around 10–20%; however, it has reached 33% to 50% with conversion therapy [5,6], which implies an increase in overall survival benefit from 12 months to 30 months over the last 20 years [7].

First-line standard treatment for mCRC patients is still a challenging issue for oncologists. Recommendations include different combinations of drugs, and doublet chemotherapeutic regimens are considered the best standard of care for mCRC. First-line chemotherapy options include fluoropyrimidine-based agents plus leucovorin and oxaliplatin (FOLFOX), or irinotecan (FOLFIRI) or capecitabine plus oxaliplatin [8].

About 60–80% of CRC shows EGFR overexpression. This fact allows the use of biologic treatments based on monoclonal antibodies (mAbs) against this receptor, which block ligand binding and inhibit the MAPK signaling pathway [9]. *KRAS* mutations negatively predict response to these inhibitors [10], and anti-VEGFR (vascular endothelial growth factor) therapies are recommended in the presence of these mutations [11]. The combination of standard chemotherapy and biologic treatment has significantly improved the outcome of mCRC patients in first- and second-line treatment [12,13]. Therefore, testing *KRAS* status is recommended in routine clinical practice for better patient management [14]. *BRAF* V600E mutation also appears to predict the lack of benefit from anti-EGFR mAbs, and it has also been considered a biomarker of poor prognosis and resistance to standard therapies [15,16,17]. Unfortunately, mCRC patients treated with these antibodies progress and develop resistance in most cases [18]. If the disease progresses, one should administer different cytotoxic drugs in order to avoid chemotherapy resistance [19,20].

*KRAS* is a factor that belongs to the GTPase RAS superfamily and triggers crucial signaling cascades, such as the RAF-MEK-ERK (MAPK) kinase cascade or PIK3CA-AKT-mTOR axis. These pathways are involved in cell growth, proliferation, differentiation or survival, and they perform a key role in tumorigenesis when they are aberrantly activated [21]. For these reasons, *KRAS* is considered one of the most important players in human cancers. Activating mutations in *KRAS* have been described in 90% of pancreatic tumors, 35% of lung cancers and 30–50% of CRCs [22]. *KRAS* mutations are found in codons 12 or 13 of exon 2 in the 90% of CRCs, especially in the phosphate-binding loop of the protein. It has been suggested that mutations at codon 12 are involved in local invasion and metastasis, whereas mutations at codon 13 could be more related to adenoma–carcinoma transition [23]. As activating *KRAS* mutations are major events that drive tumorigenesis in many types of tumors, it is currently one of the most important targets for drug development.

On the other hand, *BRAF* encodes a cytoplasmic serine–threonine kinase that acts immediately downstream of *KRAS* in the MAPK signaling pathway. Its aberrant activation enhances cell proliferation and survival and it is critical for tumorigenesis in many types of tumors [24]. Over 90% of activating *BRAF* mutations in CRC are due to a change in the nucleotide 1799 of the exon 15, which causes a thymine to adenine change, leading to a substitution of valine by glutamate. This mutation is known as *BRAF* V600E mutation [25], and has been found in 100% of hairy cell leukaemias [26], in approximately 50% of melanomas [27], in 50% of papillary thyroid cancers [28,29] or in 1–3% of non-small cell lung carcinomas [30,31]. In mCRC the incidence of *BRAF* mutations is less than 10% [15,32]; however, *BRAF* V600E mutation is considered a relevant therapeutic target for mCRC management. It has been described that *KRAS* and *BRAF* mutations are mutually exclusive in CRC, as though they are functionally redundant or incompatible, and cells with both mutations are erased [33,34]. Nevertheless, a recent study has described concomitant *KRAS* and *BRAF* mutations in CRC [35].

The role of *KRAS* and *BRAF* mutations in CRC survival and response to standard chemotherapy regimens is still controversial. Some studies support their potential application as prognosis biomarkers in CRC management [36,37,38], whereas other studies are not conclusive [39,40]. Regarding standard chemotherapy response, their potential use as predictive biomarkers is not well-established. Some articles have suggested a potential role of these mutations in standard chemotherapy response [41,42], while others have refused it [17,43]. Recently, a novel role of tumor location in the outcome of patients has been described. A more favourable outcome in left tumors than right tumors in wild-type *KRAS* CRC patients has been described [44,45].

As there is a lack of consensus in the scientific community about the role of *KRAS* and *BRAF* mutations as prognosis and predictive biomarkers for standard chemotherapy response, in this study we evaluated these mutations in a cohort of 561 patients with mCRC to provide new insights in regards to this.

## 2. Materials and Methods

### 2.1. Patients

**Figure 1:**
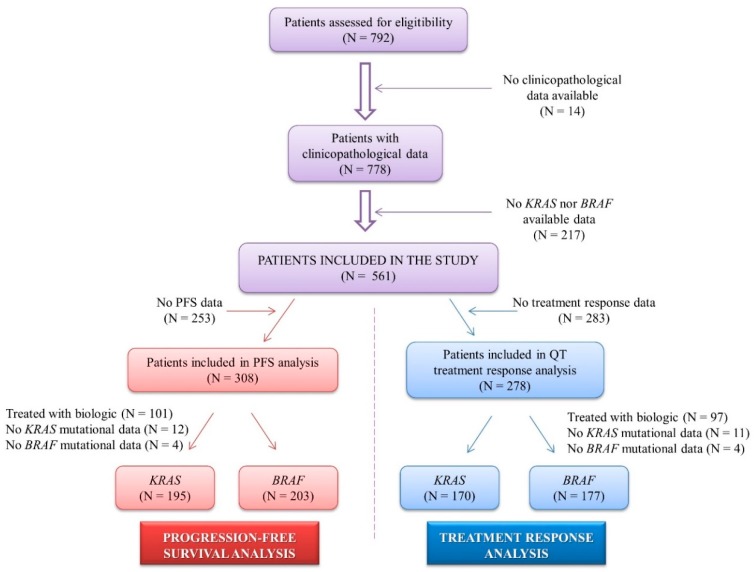
Flow chart presenting the number of patients in progression-free survival and chemotherapy response analyses performed in the study. N: number of patients.

A total of 792 patients with metastasic colorectal cancer who were diagnosed between 1984 and 2012 at the Oncology Department of the Fundacion Jimenez Diaz Hospital were assessed for eligibility. Mutational status from 217 patients could not be determined, and clinical and pathological data from 14 patients were not available; then, a total of 561 patients were included in the study. Among them, 308 patients presented progression-free survival (PFS) information, and 278 patients had complete clinical information on their chemotherapy response (Figure 1). Patients received standard chemotherapy according to standard oncology guidelines, and treatment response was evaluated following the RECIST criteria.

### 2.2. Mutational Analysis

*KRAS* and *BRAF* mutational status was performed in the Pathology Department of Fundacion Jimenez Diaz Hospital. For this, DNA was extracted with a DNA Sample Preparation Kit (Roche Diagnostics, Mannheim, Germany) according to the manufacturer’s instructions. *KRAS* mutational status of each sample was determined by Real-Time PCR with a cobas^®^ *KRAS* Mutation Test (Roche Diagnostics). *BRAF* V600E mutation testing was performed by cobas^®^ *BRAF* mutation test IVD (Roche Diagnostics).

### 2.3. Statistical Analysis

*KRAS* and *BRAF* mutational status were used as categorical variables. *KRAS* mutations in codons 12, 13 or 61 were considered as a positive *KRAS* mutation. *BRAF* mutational status was analysed as the presence or absence of a V600E mutation. Patients that achieved complete response (CR) or partial response (PR) were categorized as responders, whereas those that exhibited stable disease (SD) or progression on disease (PD) were considered non-responders. Progression-free survival was defined as the time between the dates of diagnosis and recurrence or last follow-up.

The association between *KRAS* mutations or *BRAF* V600E mutation with clinicopathological features of patients and treatment response was analysed with Chi-square test or two-tailed Fisher’s exact test. We performed survival curves for *KRAS* or *BRAF* mutations with Kaplan Meier, and statistical analysis was assessed with the log-rank test. The Cox univariate proportional hazards model was used to compare progression-free survival hazard ratios and 95% confidence intervals of *BRAF* and *KRAS* mutations with other clinical and pathological characteristics. Cox multivariate analysis was performed with those statistically significant covariates obtained from univariate analysis. *p* ≤ 0.05 was considered statistically significant. Statistical analysis was performed with the IBM SPSS program, version 20.0.

### 2.4. Ethics Statement

Clinical samples used in the study were kindly supplied from the BioBank of the Fundacion Jimenez Diaz-Universidad Autonoma de Madrid (PT13/0010/0012 and RD09/0076/00101). This study has been evaluated by the *Ethical Review Board* of the Health Research Institute of Fundacion Jimenez Diaz (act number 17/14). All patients gave written informed consent for the use of their biological samples for research purposes. Moreover, fundamental ethical principles promoted by Spain (LOPD 15/1999) and the European Union Fundamental Rights of the EU (2000/C364/01) were followed. In addition, all patients´ data were processed according to the Declaration of Helsinki (last revision 2013) and Spanish National Biomedical Research Law (14/2007, of 3 July).

## 3. Results

### 3.1. Patients Characteristics

From our institutional cohort of 792 mCRC patients, a total of 561 mCRC patients with available data of *KRAS* mutations and/or *BRAF* V600E mutations were included in the study. The median age of patients was 68 years (range 24–93 years) and most patients were male (60.1%). Among the overall population, 395 patients (70.4%) had primary tumor localization in the descending colon (left colon, sigma or rectum), whereas 158 (28.2%) were localized in the ascending colon (cecum, right colon or transverse colon). Of them, 323 patients (57.6%) presented with synchronous metastasis. Tumors cells were differentiation grade I (well-differentiated) in 15.3% of cases, 67.9% were grade II (moderately differentiated), and 9.5% grade III (poorly differentiated). Performance status (ECOG) was ≥2 in 23.9% of cases. Regarding metastatic foci, 153 patients (27.3%) showed metastasis in more than one site, while 394 of patients (70.2%) exhibited metastasis in only one site. Liver metastases were found in most of the patients (66.8%), followed by lung metastases (33.3%), or peritoneal metastases (24.2%). Furthermore, 24.4% of patients had lymph node involvement. In our study, we found that *KRAS* and *BRAF* mutations were mutually exclusive. Mutations in *KRAS* codons 12/13 were found in 46.2% of patients, whereas mutations in codon 61 were only detected in 3.4% of the cases. *BRAF* was mutated in 6.1% of patients. The overview of the clinico-pathological features of our institutional cohort of patients is given in Table 1.

Then, we aimed to verify whether *KRAS* or *BRAF* mutational status was associated with any of the clinical and pathological characteristics of the patients. In this study, the ECOG number was the only variable that was statistically associated with *KRAS* mutations (*p* = 0.005). *BRAF* V600E mutation presented a significant association with primary tumor located on the right colon (*p* < 0.0001), with synchronic disease (*p* = 0.019), and with peritoneal metastasis (*p* = 0.002), and showed a high trend towards significance with moderately and poorly differentiated tumors (differentiation grades 2 and 3, *p* = 0.098) (Table 2). These results suggest a potential implication of *KRAS* and *BRAF* mutations in the tumorigenesis of mCRC.

### 3.2. Patients That Received Biologic Treatment Showed a Better Outcome and Treatment Response

It is widely known that biologic treatment improves survival of patients; therefore, we assessed this association. In this study, cetuximab (anti-EGFR mAB) or bevacizumab (anti-VEGFR mAb) were administered to mCRC patients as biologic treatments depending on the *KRAS* mutational status of tumors. We confirmed that patients that received standard chemotherapy alone showed a worse outcome (*p* = 0.028) than those who received chemotherapy plus biologic treatment (Figure S1). Furthermore, the ratio of recurrence was higher in patients who did not receive biologic treatment (73%) compared to combination therapy (55%) (*p* = 0.002) (Table S1). The predictive role of *KRAS* and *BRAF* mutations in biologic therapies response is well understood and applied by oncologist worldwide in the clinical management of mCRC. For this reason, we focused on the association between *KRAS* and *BRAF* mutations and standard chemotherapy response in order to elucidate not only the prognosis potential of *KRAS* and *BRAF*V600E mutations but also their predictive role for standard chemotherapy response.

### 3.3. KRAS and BRAF Mutations Have An Impact on Progression-Free Survival of Patients

**Figure 2:**
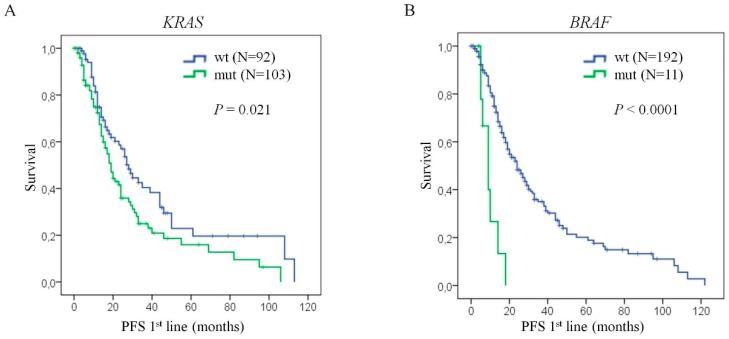
*KRAS* or *BRAF* mutations confer poor prognosis to mCRC patients. Kaplan Meier curves of progression-free survival of mCRC patients, (**A**) with *KRAS* mutations versus *KRAS* wild-type, and (**B**) with *BRAF*V600E mutations versus *BRAF* wild-type. Statistical analysis performed with log-rank test. N: number of patients; PFS: progression-free survival; wt: wild-type; mut: mutated; *P*: *p*-Value.

*KRAS* and *BRAF* mutational status was available in 195 and 203 cases, respectively, of the total number of patients who did not receive biologic treatment. *KRASBRAFKRAS* mutations were detected in 103/195 patients (53%). *KRAS* mutations were significantly associated with progression-free survival (*p* = 0.021) (Figure 2A). The median PFS was 28 months (95% CI: 22.206–33.794 months) for *KRAS* wild-type patients while patients with *KRAS* mutations has a PFS of 19 months (95% CI: 15.678–22.322 months). Concerning *BRAF* analysis, we found a mutation in *BRAF* in only 11 cases (5%). Despite the scarce number of patients with this alteration, *BRAF*V600E mutation was associated with PFS (*p* < 0.0001; Figure 2B). Here, patients with *BRAF* wild-type presented with longer median progression-free survival (median = 24 months, 95% CI: 18.678–29.322 months) in comparison to *BRAF* mutated patients (median = 9 months, 95% CI: 5.167–12.833 months). Therefore, these results confirm the implication of *KRAS* and *BRAF* mutations on patient prognosis.

Cox proportional hazards model for progression-free survival was performed to compare the prognostic potential of *KRAS* and *BRAF*V600E mutational status with the other clinico-pathological characteristics included in the study. Here, the univariate analysis revealed that patients with mutations in *KRAS* present higher risk of recurrence than wild-type patients (HR = 1.529, 95% CI: 1.058–2.211, *p* = 0.024). Moreover, *BRAF* mutated patients also showed higher risk of progression than *BRAF* wild-type (HR = 4.288, 95% CI: 2.033–9.046, *p* < 0.0001). We have also found a statistically significant association between PFS and the presence of liver metastasis (HR = 1.691, 95%CI: 1.177–2.432, *p* = 0.005). Interestingly, after multivariate analysis, these three factors remained statistically significant, not only *BRAF* (HR = 5.861, 95%CI: 2.531–13.570, *p* < 0.0001), but also *KRAS* (HR = 1.643, 95%CI: 1.110–2.431, *p* = 0.013) and liver metastasis (HR = 1.595, 95% CI: 1.086–2.343, *p* = 0.017) (Table 3). These results confirm the prognostic value of *KRAS* and *BRAF*V600E mutations for the adequate management of mCRC patients in routine clinical practice.

### 3.4. KRAS Mutation Status is Associated with a Lack of Benefit From Standard Chemotherapy

In regards to treatment regimens, a total of 278 mCRC patients were treated with standard chemotherapy as first line therapy. Among them, 97 patients were excluded from the study as they received biologic treatment regimens based on anti-EGFR or anti-VEGFR monoclonal antibodies. As a result, a total number of 170 patients with *KRAS* mutational status and 177 patients with *BRAF* mutational status were included in the study to assess the association between standard chemotherapy response and *KRAS* or *BRAF* mutation status (Figure 1). Among the *KRAS* mutated subgroup, 75/93 (81%) patients presented with stable disease or progression to standard chemotherapy compared to 48/93 (62%) patients with *KRAS* wild-type. Furthermore, only 19% of patients with a *KRAS* mutation achieved complete or partial response to treatment, while double the number of patients with *KRAS* wild-type (38%) showed complete or partial response (*p* = 0.008) (Table 4). In contrast, *BRAF* mutation did not show a clear association with treatment response, where 73% of patients with *BRAF* wild-type presented with disease recurrence compared to 78% of the *BRAF* mutated subgroup (*p* = 0.540) (Table 4). Overall, these results support the role of *KRAS* mutational status as a predictive biomarker not only for biologic treatment but also for standard chemotherapy response.

## 4. Discussion

Personalized medicine is based on the premise that patients can be stratified into subgroups according to predictive biomarkers in order to be treated with target therapies [46]. The use of monoclonal antibodies against EGFR or VEGFR in the management of mCRC has represented a substantial improvement in the outcome of these patients [47]. As a result, most mCRC patients nowadays receive biologic treatment as first-line therapy. In this study, we have observed in our cohort of patients that the combination of biologic therapy plus standard chemotherapeutic agents presents a benefit both in outcome of patients and treatment response compared to the use of standard chemotherapy alone, which goes in accordance with previous studies.

*KRAS* and *BRAF* mutation status is broadly used in the routine clinical practice for the diagnosis and treatment management in the field of CRC. *KRAS* mutation is considered a key point for treatment decisions in oncology as it can predict resistance to monoclonal antibodies against epidermal growth factor receptor (EGFR) [48], restricting the use of these therapies to *KRAS* wild-type CRC patients [10]. *BRAF* mutations also confer resistance to anti-EGFR therapies in patients with mCRC [49,50]. Biologic treatment is predominantly administered in combination with standard chemotherapy to mCRC patients. The current selection of standard chemotherapeutic treatment strategies is mostly based on the patients characteristics and the clinical, pathological and molecular features of tumors [51,52]. As there are not useful predictive biomarkers to select chemotherapeutic strategies for mCRC, it is necessary to identify new molecular markers to predict standard chemotherapy response.

Several studies have tried to elucidate the role of *KRAS* and *BRAF* mutations in cytotoxic drugs response, but the impact of the mutational status on the effectiveness of standard chemotherapy remains unclear. Furthermore, most of these studies include a low number of patients, which limits their statistical power. Lin et al. suggested *KRAS* mutation as a predictor of oxaliplatin sensitivity in vitro by downregulation of ERCC1 [53]. Indeed, a benefit of oxaliplatin-based chemotherapy for *KRAS* mutated mCRC patients has been reported [41]. A recent study suggests that patients with mutations in *KRAS* codon 12 present a significant survival advantage compared to wild-type patients treated with irinotecan-based therapy [54]. However, other studies reported that mutation status of *KRAS* did not impact on irinotecan or oxaliplatin response [17,55]. Other studies reported a lack of benefit from chemotherapy for those *KRAS* mutated mCRC patients [43,56]. Cui et al. reported in a meta-analysis that patients with *BRAF* mutations showed worse objective response rates than wild-type patients treated with standard chemotherapy [42]. Other studies suggested that the predictive role of *BRAF* mutations is not significant in standard chemotherapy response [17,57]. Therefore, there is an absence of evidence to conclude that *KRAS* or *BRAF* mutated patients are less likely to obtain a benefit from standard chemotherapeutic strategies.

In our study, we provide new insight on the role of *KRAS* and *BRAF* mutations, focused on the prediction of response to adjuvant standard chemotherapy alone based mainly on oxaliplatin, irinotecan and fluoropyrimidines. Moreover, the fact that all patients have been treated at a single institution makes this study highly homogeneous. Here, we have observed that *KRAS* mutations can predict resistance to standard chemotherapy (*p* = 0.008). Thus, patients showed stable disease or relapse in 81% (N = 75) of cases with *KRAS* mutation, compared to 62% (N = 48) of *KRAS* wild-type patients. Unfortunately, we could not stratify our cohort of patients regarding the specific chemotherapeutic agents used because of the low number of cases in some of the subgroups.

The use of biologic treatment could influence the prognosis impact of *KRAS* and *BRAF* mutations in the outcome of patients. Indeed, the role of *KRAS* as a prognostic biomarker in CRC is still rather controversial. *KRAS* mutations have been associated with a worse prognosis in several reports [58,59,60,61]. However, there are other several articles that have failed to show a role of *KRAS* mutations in the outcome of patients [62,63,64,65]. For this reason, we aimed to analyse the prognostic potential of *KRAS* and *BRAF* mutations in a subset of patients that did not receive biologic therapy along the study period and only received standard chemotherapy as first-line treatment.

Here, we provide evidence on the impact of *KRAS* mutations on the time to progression of patients. Nevertheless, it would be interesting to analyse the role of the specific mutations in the *KRAS* gene. For instance, Jones et al. found that different mutations in *KRAS* codon 12 have different impacts on survival, especially G12C and G12V, which remained significant among the five most common mutations [58]. The poorer prognosis role of these specific mutations in *KRAS* was in codon 12, which was confirmed recently by different authors [66,67]. Mutations in codon 146 are also found in mCRC patients but at a low frequency [68,69]. Nevertheless, their role in mCRC prognosis and treatment response is still unknown due to the limited statistical power of these analyses, and large-scale trials are needed [70]. The role of *KRAS* mutation status in other types of tumors has also been studied, e.g., *KRAS* mutation is a poor prognostic factor and erlotinib or gefitinib resistance biomarker in non-small cell lung cancer [71,72].

The *BRAF* mutation was also significantly associated with poor outcome in our cohort of patients. Indeed, Cox proportional hazards model confirmed the association between shorter progression-free survival and *KRAS* mutation, *BRAF* mutation and the presence of liver metastasis than other clinicopathological variables. This worst prognosis impact is consistent with previous publications about mCRC [37,73,74,75]. Furthermore, the *BRAF* V600E mutation has also been associated with a reduced survival in other types of tumors like melanoma [76] or papillary thyroid cancer [77].

In addition, we have found an association between *KRAS* or *BRAF* mutation status and the differentiation grade of tumors, which suggests a role of these activating mutations in tumor development and progression. *KRAS* mutation was also associated with a higher performance status. Interestingly, most patients with a *BRAF* mutation (N = 26, 76%) also exhibited synchronic metastatic disease. This observation suggests that *BRAF* V600E mutation is an early event that contributes to tumorigenesis of mCRC. Indeed, the *BRAF* mutation was positively associated with the presence of peritoneal metastases (*p* = 0.002), and revealed a high trend towards significance with lymph node affection (*p* = 0.054); however, it is unlikely to be associated with lung metastases (*p* = 0.200). These results go in accordance to those of Tran el al. regarding localization of metastases. These authors reported 46% vs. 24% for peritoneal metastasis when comparing *BRAF* mutated vs. *BRAF* wild-type respectively, 53% vs. 38% for lymph nodes metastases, and 35% vs. 49% for lung metastasis [37]. In our cohort, 47% of *BRAF* mutated patients showed peritoneal metastases vs. 23% *BRAF* wild-type patients, 38% show lymph nodes metastases vs. 24%, and 24% vs. 34% show lung metastases respectively. In both studies, *BRAF* mutated patients are more likely to present with peritoneal metastases and lymph node affection, and the *BRAF* mutation does not seem to confer tropism to lungs. It has also been reported that *BRAF* wild-type tumors are usually localized in the left side of the colon, while *BRAF* mutations are associated with the right side of the colon [78,79]. In our cohort, 75% of *BRAF* wild-type tumors were localised in the left colon, whereas 79% of *BRAF* mutated tumors were found predominantly in the right side, which goes in accordance with the literature. Therefore, these results contribute to the understanding of the role of the *BRAF* mutation in mCRC management, where *BRAF* is providing new treatment strategies based on molecular targeted therapy [80].

## 5. Conclusions

Most published articles analyse the impact of *KRAS* and *BRAF* mutations in mCRC patients that received standard chemotherapy in combination with biologic treatment. Moreover, their prognostic potential is rather controversial. In the present study, we have provided new insights into the roles of *KRAS* and *BRAF* mutations, and we propose their use as prognostic and predictive biomarkers for standard chemotherapy response in the mCRC scenario.

## Figures and Tables

**Table 1:** Clinico-pathological characteristics of CRC patients from our institutional set included in the study.

Characteristics	N (%)	Characteristics	N (%)
**Median age (range)**	68 years (24–93)	**1st line treatment response**	
**Gender**		CR or PR	94 (16.8%)
Female	224 (39.9%)	SD or PD	186 (33.2%)
Male	337 (60.1%)	N/A	281 (50.0%)
**Localization of primary tumor**		**Number of metastatic sites**	
Right colon	158 (28.2%)	1	394 (70.2%)
Left colon	395 (70.4%)	>1	153 (27.3%)
N/A	8 (1.4%)	N/A	14 (2.5%)
**Metastatic pattern**		**Liver metastasis**	
Metachronous	237 (42.2%)	No	185 (33.0%)
Synchronous	323 (57.6%)	Yes	375 (66.8%)
N/A	1 (0.2%)	N/A	1 (0.2%)
**Differentiation grade**		**Lung metastasis**	
G1	86 (15.3%)	No	372 (66.3%)
G2	381 (67.9%)	Yes	187 (33.3%)
G3	53 (9.5%)	N/A	2 (0.4%)
N/A	41 (7.3%)	**Lymph nodes metastasis**	
**ECOG**		No	423 (75.4%)
0	167 (29.8%)	Yes	137 (24.4%)
1	248 (44.2%)	N/A	1 (0.2%)
2	88 (15.7%)	**Peritoneal metastasis**	
3	46 (8.2%)	No	424 (75.6%)
N/A	12 (2.1%)	Yes	136 (24.2%)
**Metastatic 1st backbone**		N/A	1 (0.2%)
Oxaliplatin-based	163 (29.1%)	***KRAS* mutational status**	
Irinotecan-based	74 (13.2%)	Wild-type	254 (45.3%)
Fluoropyrimidines	70 (12.5%)	Mutated in 12/13	259 (46.2%)
Other	12 (2.1%)	Mutated in 61	19 (3.4%)
None	242 (43.1%)	N/A	29 (5.1%)
**Metastatic 1st biologic**		***BRAF* mutational status**	
None	458 (81.6%)	Wild-type	519 (92.5%)
Cetuximab	39 (7.0%)	V600E Mutated	34 (6.1%)
Bevacizumab	64 (11.4%)	N/A	8 (1.4%)

N: number of patients; CR: complete response; PR: partial response; SD: stable disease; PD: progressive disease; ECOG: Eastern Cooperative Oncology Group performance status scale; N/A: not available.

**Table 2:** Statistical association between *KRAS* and *BRAF* mutational status with clinico-pathological features of the patients.

Clinico-Pathological Feature	*KRAS* Wt	*KRAS* Mut	*p*-Value	*BRAF* Wt	*BRAF* Mut	*p*-Value
**Age (median, range)**	67.0 (27–93)	69.0 (24–90)		68.5 (24–93)	63.5 (33–85)	
**Gender**			0.123			0.610
Male	161 (63%)	158 (57%)		313 (60%)	19 (56%)	
Female	93 (37%)	120 (43%)		206 (40%)	15 (44%)	
**Tumor location**			0.179			0.000
Right colon	65 (26%)	84 (31%)		130 (25%)	27 (79%)	
Left colon	188 (74%)	187 (69%)		381 (75%)	7 (21%)	
**Metastatic disease**			0.763			0.019
Metachronous	104 (41%)	117 (42%)		228 (44%)	8 (24%)	
Synchronous	150 (59%)	160 (58%)		290 (56%)	26 (76%)	
**Grade**			0.113			0.098
G1	31 (13%)	49 (19%)		83 (17%)	2 (7%)	
G2 + G3	199 (87%)	212 (81%)		400 (83%)	28 (93%)	
**ECOG**			0.005			0.771
0/1	205 (82%)	193 (71%)		384 (76%)	25 (74%)	
2/3	45 (18%)	77 (29%)		123 (24%)	9 (26%)	
**Liver metastasis**			0.105			0.161
No	91 (36%)	81 (29%)		168 (32%)	15 (44%)	
Yes	163 (64%)	196 (71%)		350 (68%)	19 (56%)	
**Lung metastasis**			0.400			0.200
No	171 (68%)	179 (64%)		340 (66%)	26 (76%)	
Yes	81 (32%)	99 (36%)		177 (34%)	8 (24%)	
**Lymph nodes metastasis**			0.897			0.054
No	194 (76%)	210 (76%)		396 (76%)	21 (62%)	
Yes	60 (24%)	67 (24%)		122 (24%)	13 (38%)	
**Peritoneal metastasis**			0.446			0.002
No	190 (75%)	215 (78%)		399 (77%)	18 (53%)	
Yes	64 (25%)	62 (22%)		119 (23%)	16 (47%)	
**Number of metastatic sites**			0.564			0.483
1	180 (73%)	192 (71%)		366 (72%)	22 (67%)	
>1	67 (27%)	80 (29%)		140 (28%)	11 (33%)	

N: number of patients; ECOG: Eastern Cooperative Oncology Group as performance status scale; wt: wild-type; mut: mutated.

**Table 3:** Uni- and multivariate proportional hazard model of *KRAS* and *BRAF,* and other clinico-pathological variables in the progression-free survival of mCRC patients.

	Univariate PFS (95%CI)			
	HR	Lower	Upper	*p*-Value
**Age** (< vs. >68 years)	1.137	0.802	1.612	0.470
**Gender** (Female vs. Male)	1.166	0.820	1.659	0.393
**Localization** (Right colon vs. Left colon)	1.245	0.840	1.844	0.275
**Grade**				0.757
G1	1.000			
G2	0.859	0.311	2.370	0.769
G3	1.056	0.428	2.603	0.906
**ECOG** (0/1 vs. 2/3)	1.331	0.775	2.284	0.300
**Metastatic 1st backbone**				0.301
Oxaliplatin-based	1.000			
Irinotecan-based	0.914	0.615	1.360	0.659
Fluoropyrimidines	1.295	0.818	2.051	0.269
**Liver metastasis** (No vs. Yes)	1.691	1.177	2.432	0.005
**Lung metastasis** (No vs. Yes)	1.320	0.924	1.887	0.128
**Lymph nodes metastasis** (No vs. Yes)	1.178	0.809	1.716	0.393
**Peritoneal metastasis** (No vs. Yes)	1.333	0.892	1.991	0.161
**Number of metastasis sites** (1 vs. >1)	1.387	0.931	2.067	0.108
***KRAS*mutation** (No vs. Yes)	1.529	1.058	2.211	0.024
***BRAF*mutation** (No vs. Yes)	4.288	2.033	9.046	0.000
	**Multivariate PFS (95% CI)**			
**Liver metastasis** (No vs. Yes)	1.595	1.086	2.343	0.017
***KRAS*mutation** (No vs. Yes)	1.643	1.110	2.431	0.013
***BRAF*mutation** (No vs. Yes)	5.861	2.531	13.570	0.000

PFS: progression-free survival; HR: hazard ratio; CI: confidence interval; vs: versus; ECOG: Eastern Cooperative Oncology Group performance status scale.

**Table 4:** Statistical association between *KRAS* and *BRAF* mutational status and treatment response to standard chemotherapy.

	CR + PR	SD + PD	*p*-Value
***KRAS* wild-type**	29 (38%)	48 (62%)	
***KRAS* mutated**	18 (19%)	75 (81%)	
			0.008
***BRAF* wild-type**	46 (27%)	122 (73%)	
***BRAF* mutated**	2 (22%)	7 (78%)	
			0.540

CR: complete response; PR: partial response; SD: stable disease; PD: progressive disease.

## Supplementary Materials

The following are available online at https://www.mdpi.com/2073-4409/9/1/219/s1, Figure S1: Patient treated with biologic therapy presented longer progression-free survival, Table S1: Treatment response of patients that received standard chemotherapeutic alone vs. standard chemotherapy plus biologic treatment.
