# Supplementary material for: KRAS and BRAF Mutations as Prognostic and Predictive Biomarkers for Standard Chemotherapy Response in Metastatic Colorectal Cancer: A Single Institutional Study
Source: Cells. 2020 Jan 15;9(1):219. doi: 10.3390/cells9010219 (PMC7016634; doi:10.3390/cells9010219)
Supplement: Supplementary file 1 [file cells-09-00219-s001.zip › cells-680172. supplementary/Table 1. Clinicopathological characteristics descriptive.docx]

**Table 1.** Clinico-pathological characteristics of CRC patients from our institutional set included in the study

|  |  | | |  | |  |  |  | |  |  |
| --- | --- | --- | --- | --- | --- | --- | --- | --- | --- | --- | --- |
| **Characteristics** | | | | **N (%)** | |  | **Characteristics** | | | **N (%)** |  |
| **Median age (range)** | | |  | 68 years (24-93) | |  | **1^st^ line treatment response** | | |  |  |
| **Gender** | | |  |  | |  | CR or PR | | | 94 (16.8%) |  |
| Female | | | | 224 (39.9%) | |  | SD or PD | |  | 186 (33.2%) |  |
| Male | |  | | 337 (60.1%) | |  | N/A | |  | 281 (50.0%) |  |
| **Localization of primary tumor** | | |  |  | |  | **Number of metastatic sites** | |  |  |  |
| Right colon |  | | | 158 (28.2%) | |  | 1 | | | 394 (70.2%) |  |
| Left colon | | | | | 395 (70.4%) |  | >1 | |  | 153 (27.3%) |  |
| N/A | | |  | 8 (1.4%) | |  | N/A | |  | 14 (2.5%) |  |
| **Metastatic pattern** | | |  |  | |  | **Liver metastasis** | |  |  |  |
| Metachronous | | |  | 237 (42.2%) | |  | No | | | 185 (33.0%) |  |
| Synchronous | | |  | 323 (57.6%) | |  | Yes | | | 375 (66.8%) |  |
| N/A | | |  | 1 (0.2%) | |  | N/A | | | 1 (0.2%) |  |
| **Differentiation grade** | | |  |  | |  | **Lung metastasis** | |  |  |  |
| G1 | | |  | 86 (15.3%) | |  | No | |  | 372 (66.3%) |  |
| G2 | | |  | 381 (67.9%) | |  | Yes | |  | 187 (33.3%) |  |
| G3 | | |  | 53 (9.5%) | |  | N/A | |  | 2 (0.4%) |  |
| N/A | | |  | 41 (7.3%) | |  | **Lymph nodes metastasis** | |  |  |  |
| **ECOG** | | |  |  | |  | No | |  | 423 (75.4%) |  |
| 0 | | |  | 167 (29.8%) | |  | Yes | |  | 137 (24.4%) |  |
| 1 | | |  | 248 (44.2%) | |  | N/A | |  | 1 (0.2%) |  |
| 2 | | |  | 88 (15.7%) | |  | **Peritoneal metastasis** | |  |  |  |
| 3 | | |  | 46 (8.2%) | |  | No | |  | 424 (75.6%) |  |
| N/A | | |  | 12 (2.1%) | |  | Yes | |  | 136 (24.2%) |  |
| **Metastatic 1^st^ backbone** | | |  |  | |  | N/A | |  | 1 (0.2%) |  |
| Oxaliplatin-based | | |  | 163 (29.1%) | |  | ***KRAS* mutational status** | | |  |  |
| Irinotecan-based | | |  | 74 (13.2%) | |  | Wild-type |  | | 254 (45.3%) |  |
| Fluoropyrimidines | | |  | 70 (12.5%) | |  | Mutated in 12/13 |  | | 259 (46.2%) |  |
| Other | | |  | 12 (2.1%) | |  | Mutated in 61 |  | | 19 (3.4%) |  |
| None | | |  | 242 (43.1%) | |  | N/A |  | | 29 (5.1%) |  |
| **Metastatic 1^st^ biologic** | | |  |  | |  | ***BRAF* mutational status** |  | |  |  |
| None | | |  | 458 (81.6%) | |  | Wild-type |  | | 519 (92.5%) |  |
| Cetuximab | | |  | 39 (7.0%) | |  | V600E Mutated |  | | 34 (6.1%) |  |
| Bevacizumab | | |  | 64 (11.4%) | |  | N/A |  | | 8 (1.4%) |  |

N: number of patients; CR: complete response; PR: partial response; SD: stable disease; PD: progressive disease; ECOG: Eastern Cooperative Oncology Group performance status scale; N/A: not available.
