# Supplementary material for: KRAS and BRAF Mutations as Prognostic and Predictive Biomarkers for Standard Chemotherapy Response in Metastatic Colorectal Cancer: A Single Institutional Study
Source: Cells. 2020 Jan 15;9(1):219. doi: 10.3390/cells9010219 (PMC7016634; doi:10.3390/cells9010219)
Supplement: Supplementary file 1 [file cells-09-00219-s001.zip › cells-680172. supplementary/Table 2. Crosstab todos pacientes mutvswt.docx]

**Table 2.** Statistical association between *KRAS* and *BRAF* mutational status with clinico-pathological features of the patients

| **Clinico-pathological**  **feature** | ***KRAS* wt** | ***KRAS* mut** | ***P*-value** |  | ***BRAF* wt** | ***BRAF* mut** | ***P*-value** |
| --- | --- | --- | --- | --- | --- | --- | --- |
|  |  |  |  |  |  |  |  |
| **Age (median, range)** | 67.0  (27-93) | 69.0  (24-90) |  |  | 68.5  (24-93) | 63.5  (33-85) |  |
|  |  |  |  |  |  |  |  |
| **Gender** |  |  | 0.123 |  |  |  | 0.610 |
| Male | 161 (63%) | 158 (57%) |  |  | 313 (60%) | 19 (56%) |  |
| Female | 93 (37%) | 120 (43%) |  |  | 206 (40%) | 15 (44%) |  |
|  |  |  |  |  |  |  |  |
| **Tumour location** |  |  | 0.179 |  |  |  | 0.000 |
| Right side | 65 (26%) | 84 (31%) |  |  | 130 (25%) | 27 (79%) |  |
| Left side | 188 (74%) | 187 (69%) |  |  | 381 (75%) | 7 (21%) |  |
|  |  |  |  |  |  |  |  |
| **Metastatic disease** |  |  | 0.763 |  |  |  | 0.019 |
| Metachronous | 104 (41%) | 117 (42%) |  |  | 228 (44%) | 8 (24%) |  |
| Synchronous | 150 (59%) | 160 (58%) |  |  | 290 (56%) | 26 (76%) |  |
|  |  |  |  |  |  |  |  |
| **Grade** |  |  | 0.113 |  |  |  | 0.098 |
| G1 | 31 (13%) | 49 (19%) |  |  | 83 (17%) | 2 (7%) |  |
| G2+G3 | 199 (87%) | 212 (81%) |  |  | 400 (83%) | 28 (93%) |  |
|  |  |  |  |  |  |  |  |
| **ECOG** |  |  | 0.005 |  |  |  | 0.771 |
| 0/1 | 205 (82%) | 193 (71%) |  |  | 384 (76%) | 25 (74%) |  |
| 2/3 | 45 (18%) | 77 (29%) |  |  | 123 (24%) | 9 (26%) |  |
|  |  |  |  |  |  |  |  |
| **Liver metastasis** |  |  | 0.105 |  |  |  | 0.161 |
| No | 91 (36%) | 81 (29%) |  |  | 168 (32%) | 15 (44%) |  |
| Yes | 163 (64%) | 196 (71%) |  |  | 350 (68%) | 19 (56%) |  |
|  |  |  |  |  |  |  |  |
| **Lung metastasis** |  |  | 0.400 |  |  |  | 0.200 |
| No | 171 (68%) | 179 (64%) |  |  | 340 (66%) | 26 (76%) |  |
| Yes | 81 (32%) | 99 (36%) |  |  | 177 (34%) | 8 (24%) |  |
|  |  |  |  |  |  |  |  |
| **Lymph nodes metastasis** |  |  | 0.897 |  |  |  | 0.054 |
| No | 194 (76%) | 210 (76%) |  |  | 396 (76%) | 21 (62%) |  |
| Yes | 60 (24%) | 67 (24%) |  |  | 122 (24%) | 13 (38%) |  |
|  |  |  |  |  |  |  |  |
| **Peritoneal metastasis** |  |  | 0.446 |  |  |  | 0.002 |
| No | 190 (75%) | 215 (78%) |  |  | 399 (77%) | 18 (53%) |  |
| Yes | 64 (25%) | 62 (22%) |  |  | 119 (23%) | 16 (47%) |  |
|  |  |  |  |  |  |  |  |
| **Number of metastatic sites** |  |  | 0.564 |  |  |  | 0.483 |
| 1 | 180 (73%) | 192 (71%) |  |  | 366 (72%) | 22 (67%) |  |
| >1 | 67 (27%) | 80 (29%) |  |  | 140 (28%) | 11 (33%) |  |
|  |  |  |  |  |  |  |  |

N: number of patients; ECOG: Eastern Cooperative Oncology Group performance status scale; wt: wild-type; mut: mutated.
