# Supplementary material for: KRAS and BRAF Mutations as Prognostic and Predictive Biomarkers for Standard Chemotherapy Response in Metastatic Colorectal Cancer: A Single Institutional Study
Source: Cells. 2020 Jan 15;9(1):219. doi: 10.3390/cells9010219 (PMC7016634; doi:10.3390/cells9010219)
Supplement: Supplementary file 1 [file cells-09-00219-s001.zip › cells-680172. supplementary/Table 3. BRAF COX sin biologico.docx]

**Table 3.** Uni- and multivariate proportional hazard model of *KRAS* and *BRAF,* and other clinical variables on progression-free survival of mCRC patients.

|  | **Univariate PFS (95%CI)** | | | |
| --- | --- | --- | --- | --- |
|  | **HR** | **Lower** | **Upper** | ***P-*Value** |
| **Age** (< *vs* > 68 years) | 1.137 | 0.802 | 1.612 | 0.470 |
| **Gender** (Female *vs* Male) | 1.166 | 0.820 | 1.659 | 0.393 |
| **Localization** (Right colon *vs* Left colon) | 1.245 | 0.840 | 1.844 | 0.275 |
| **Grade** |  |  |  | 0.757 |
| G1 | 1.000 |  |  |  |
| G2 | 0.859 | 0.311 | 2.370 | 0.769 |
| G3 | 1.056 | 0.428 | 2.603 | 0.906 |
| **ECOG** (0/1 *vs* 2/3) | 1.331 | 0.775 | 2.284 | 0.300 |
| **Metastatic 1st backbone** |  |  |  | 0.301 |
| Oxaliplatin-based | 1.000 |  |  |  |
| Irinotecan-based | 0.914 | 0.615 | 1.360 | 0.659 |
| Fluoropyrimidines | 1.295 | 0.818 | 2.051 | 0.269 |
| **Liver metastasis** (No *vs* Yes) | 1.691 | 1.177 | 2.432 | 0.005 |
| **Lung metastasis** (No *vs* Yes) | 1.320 | 0.924 | 1.887 | 0.128 |
| **Lymph nodes metastasis** (No *vs* Yes) | 1.178 | 0.809 | 1.716 | 0.393 |
| **Peritoneal metastasis** (No *vs* Yes) | 1.333 | 0.892 | 1.991 | 0.161 |
| **Number of metastasis sites** (1 *vs* >1) | 1.387 | 0.931 | 2.067 | 0.108 |
| ***KRAS* mutation** (No *vs* Yes) | 1.529 | 1.058 | 2.211 | 0.024 |
| ***BRAF* mutation** (No *vs* Yes) | 4.288 | 2.033 | 9.046 | 0.000 |
|  | **Multivariate PFS (95% CI)** | | | |
| **Liver metastasis** (No *vs* Yes) | 1.595 | 1.086 | 2.343 | 0.017 |
| ***KRAS* mutation** (No *vs* Yes) | 1.643 | 1.110 | 2.431 | 0.013 |
| ***BRAF* mutation** (No *vs* Yes) | 5.861 | 2.531 | 13.570 | 0.000 |

PFS: progression-free survival; HR: hazard ratio; CI: confidence interval; *vs*: versus; ECOG: Eastern Cooperative Oncology Group performance status scale.
