# Supplementary material for: KRAS and BRAF Mutations as Prognostic and Predictive Biomarkers for Standard Chemotherapy Response in Metastatic Colorectal Cancer: A Single Institutional Study
Source: Cells. 2020 Jan 15;9(1):219. doi: 10.3390/cells9010219 (PMC7016634; doi:10.3390/cells9010219)
Supplement: Supplementary file 1 [file cells-09-00219-s001.zip › cells-680172. supplementary/Table 4. Treatment response sinbiolog.docx]

**Table 4.** Statistical association between with *KRAS* and *BRAF* mutational status and treatment response to standard chemotherapy.

|  | **CR + PR** | **SD + PD** | ***P*-Value** |
| --- | --- | --- | --- |
| ***KRAS* wild-type** | 29 (38%) | 48 (62%) |  |
| ***KRAS* mutated** | 18 (19%) | 75 (81%) |  |
|  |  |  | 0.008 |
| ***BRAF* wild-type** | 46 (27%) | 122 (73%) |  |
| ***BRAF* mutated** | 2 (22%) | 7 (78%) |  |
|  |  |  | 0.540 |

CR: complete response; PR: partial response; SD: stable disease; PD: progressive disease
