# Supplementary material for: KRAS and BRAF Mutations as Prognostic and Predictive Biomarkers for Standard Chemotherapy Response in Metastatic Colorectal Cancer: A Single Institutional Study
Source: Cells. 2020 Jan 15;9(1):219. doi: 10.3390/cells9010219 (PMC7016634; doi:10.3390/cells9010219)
Supplement: Supplementary file 1 [file cells-09-00219-s001.zip › cells-680172. supplementary/Table supplementary 1.docx]

**Table supplementary 1.** Treatment response of patients that received standard chemotherapeutic alone *vs*. standard chemotherapy plus biologic treatment.

|  | **CR + PR** | **SD + PD** | ***P*-Value** |
| --- | --- | --- | --- |
| **CT** | 49 (27%) | 132 (73%) |  |
| **CT + Biologic** | 44 (45%) | 53 (55%) |  |
|  |  |  | 0.002 |

CR: complete response; PR: partial response; SD: stable disease; PD: progressive disease; CT: standard chemotherapy; Biologic: biologic treatment
