# Supplementary figures and images for: KRAS and BRAF Mutations as Prognostic and Predictive Biomarkers for Standard Chemotherapy Response in Metastatic Colorectal Cancer: A Single Institutional Study
Source: Cells. 2020 Jan 15;9(1):219. doi: 10.3390/cells9010219 (PMC7016634; doi:10.3390/cells9010219)

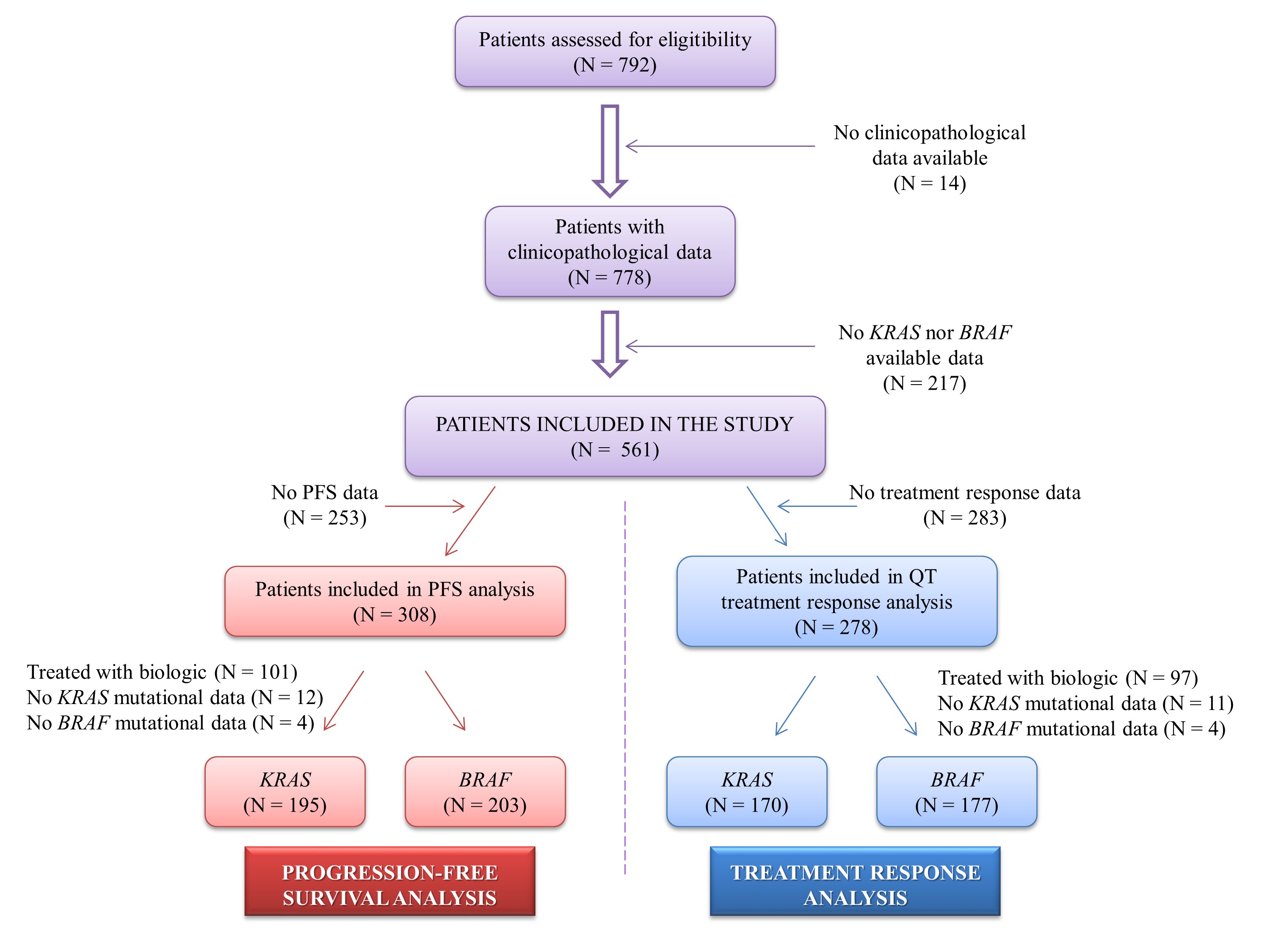

Supplement: Supplementary file 1 [file cells-09-00219-s001.zip › cells-680172. supplementary/Figure 1.jpg]

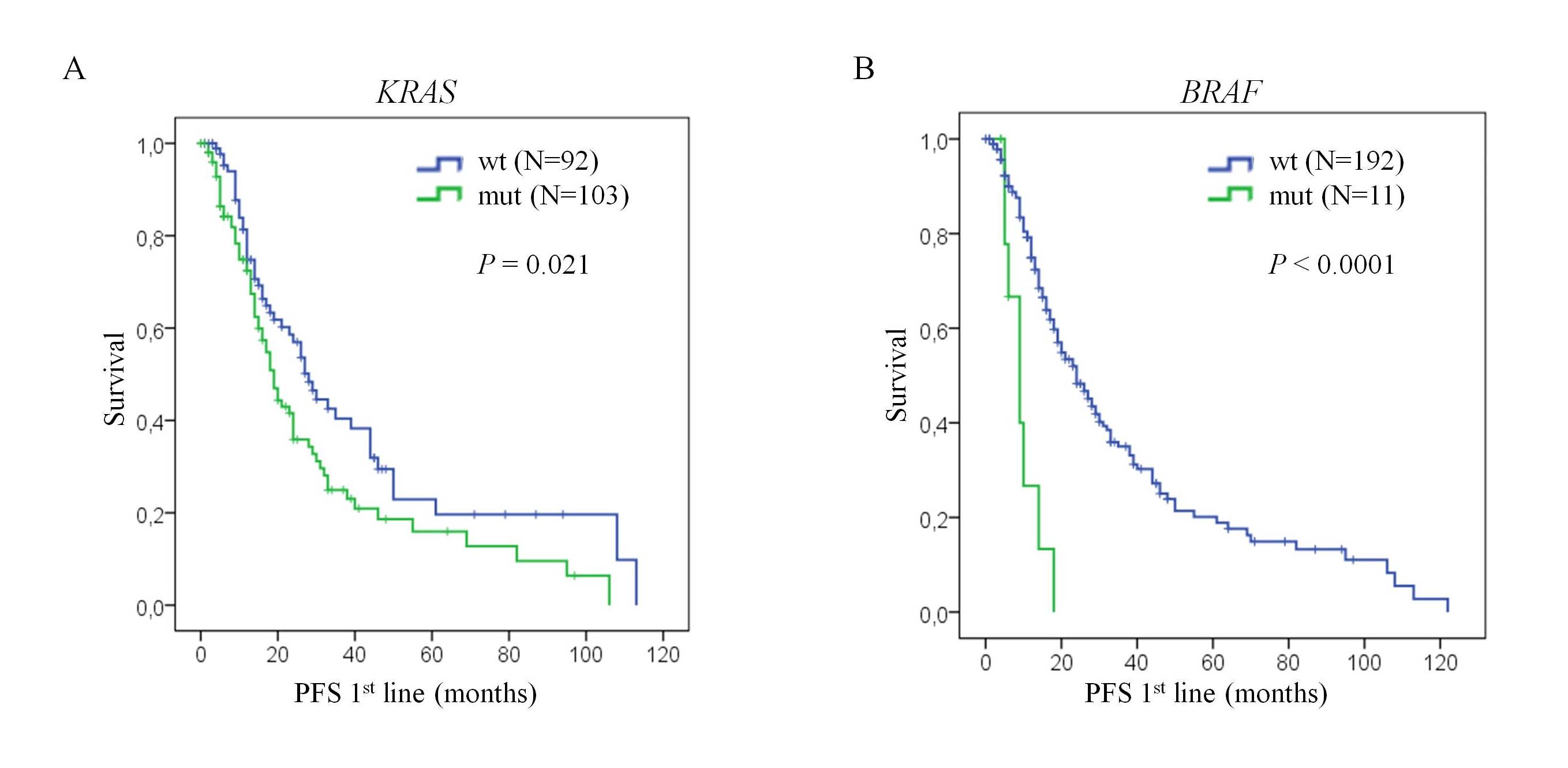

Supplement: Supplementary file 1 [file cells-09-00219-s001.zip › cells-680172. supplementary/Figure 2.jpg]

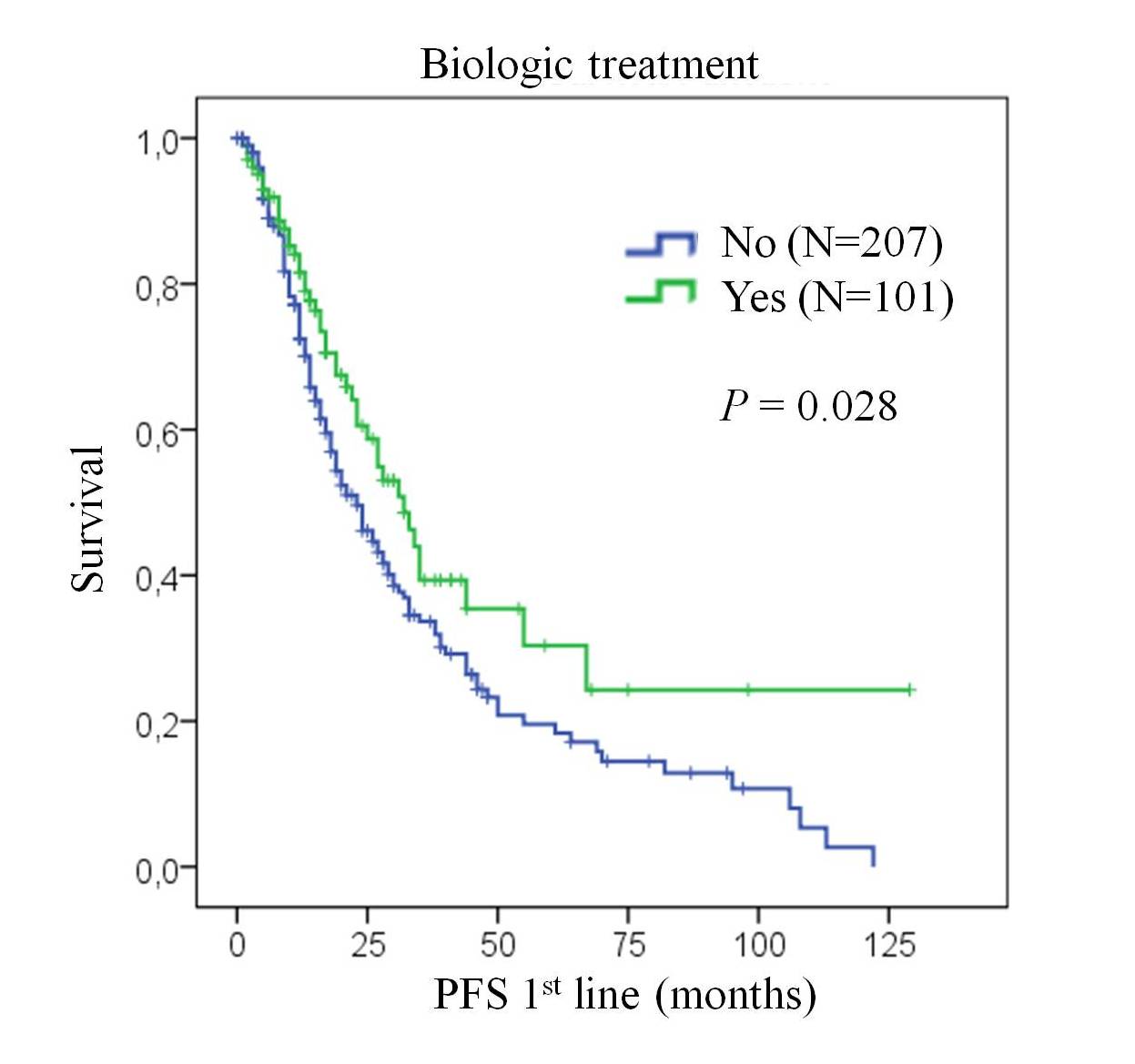

Supplement: Supplementary file 1 [file cells-09-00219-s001.zip › cells-680172. supplementary/Figure supplementary 1.jpg]
